# Association Between Breastfeeding and Child Stunting in Mexico

**DOI:** 10.5334/aogh.2836

**Published:** 2020-11-17

**Authors:** Ana Paola Campos, Mireya Vilar-Compte, Summer Sherburne Hawkins

**Affiliations:** 1 Boston College School of Social Work, Chestnut Hill, Massachusetts, US; 2 EQUIDE Research Institute, Iberoamericana University, Mexico City, MX

## Abstract

**Background:**

Globally, the prevalence of child stunting has been decreasing over the past decades. However, in low- and middle-income countries such as Mexico, stunting is still the most prevalent form of undernutrition affecting a large number of children in the most vulnerable conditions. Breastfeeding has been identified as one of the key affordable and modifiable maternal health behaviors protecting against child stunting.

**Objective:**

To examine the association between breastfeeding (defined as never breastfed, any breastfeeding for <6 months, and any breastfeeding for ≥6 months) and other individual-, household-, and area-level factors with child stunting (defined as length/height-for-age-z-score for sex under –2 standard deviations of the World Health Organization child growth standards’ median) in Mexico.

**Methods:**

Secondary data analysis using the 2012 Mexican Health and Nutrition Survey, which allowed representativeness of rural and urban areas at national level and among 4 regions in Mexico. Our subset included data on 2,089 singleton Mexican children aged 6–35 months with information on previously identified risk and protective factors for stunting. We conducted fixed- and mixed-effects logistic regression models sequentially controlling for each level of factors.

**Findings:**

Overall, 12.3% of children were stunted and 71.1% were breastfed for ≥6 months. Any breastfeeding and being female were consistent protective factors against child stunting across all models. In contrast, child low birthweight, maternal short stature, higher number of children aged <5 years per household, and moderate to severe food insecurity were consistent risk factors for child stunting across all models.

**Conclusions:**

According to our findings, efforts to reduce child stunting in Mexico should include prenatal strategies aiming to prevent low birthweight offspring particularly among short-stature women, moderate to severe food insecure households, families with a higher number of children aged <5 years, and indigenous communities. Postnatal components should include multilevel strategies to support breastfeeding.

## Introduction

Metabolic, social, and environmental risk factors during the first 1,000 days of life (conception through the first 2 years) and beyond can lead to child undernutrition [1,2,3]. The United Nations Children’s Fund (UNICEF) and the World Health Organization (WHO) estimate that undernutrition contributes to nearly half of all deaths for children aged <5 years globally [4]. Stunting is the most prevalent form of child undernutrition and is identified by measuring children’s length or height (recumbent length for children aged <2 years and standing height for those aged ≥2 years). Stunting is defined as length/height-for-age, for sex, under –2 standard deviations (SD) of the WHO child growth standards median referred to as LfA-z-score, meaning that children’s length/height is too low for their age and sex [4]. Stunting often begins in the uterus and continues for at least the first 2 years of life [2]. Child stunting remains a challenge especially in low- and middle-income countries (LMICs) in which children are at higher risk for undernutrition [4]. In LMICs, which include low-, lower-middle-, and upper-middle-income countries, child stunting has been strongly associated with later-life cognitive and metabolic disorders affecting the economic potential of individuals, households, and societies across the life span [5,6,7]. The first 1,000 days of life and beyond are a critical period to intervene and prevent stunting in order to achieve short- and long-term healthy linear growth and body weight trajectories [3]. Therefore, it is relevant to assess stunting during this timeframe and to identify its underlying pathological mechanisms.

Worldwide, the estimated prevalence of stunting in children aged <5 years has been declining over the past few decades (39.3% in 1990 versus 21.9% in 2018) [4]. However, the number of children affected by stunting (around 149 million in 2018) and its long-term consequences are still considerable, especially in LMICs [1,2,4,8]. In Mexico, an upper-middle-income country included within the LMICs category [9], the national prevalence of child stunting in this same age bracket has also decreased over the past few decades (26.9% in 1988 versus 13.6% in 2012) yet remained 2.2 percentage points higher than the aggregate prevalence for Latin American and Caribbean countries in 2012 (11.4%) [10,11], As previously reported, stunting is most prevalent among indigenous population, in the southern rural region, and in marginalized communities in Mexico [10]. Although the decreasing trend is encouraging, Mexico has yet to increase efforts to reduce health, social, and economic disparities and contribute to reach the 2025 WHO’s target to reduce the prevalence of stunting by 40% globally [12], as well as contribute to the United Nation’s sustainable development goals (SDGs) to end all forms of child malnutrition by 2030 [13].

Stunting has been associated with increased child morbidity and mortality, lower educational performance during childhood, and later-life reduced socioeconomic status (SES) and increased metabolic diseases [5,7,14]. While stunted children may catch up in linear growth during the first 2 years of life, cognitive damage seems to persist past this early period [15,16]. For instance, at age 5, children who experienced early stunting performed significantly worse on cognitive tests when compared with children who did not experience early stunting, which has serious implications for schooling indicators, such as readiness and achievement [5]. Stunting is considered a marker for social and health inequalities and helps identify underserved communities in which short stature is the norm [2]. In the latter, stunting is a pervasive process through which there is an intergenerational effect on linear growth, meaning that short stature women who were stunted during their infancy tend to have stunted offspring carried on from intrauterine growth restriction (IUGR), perpetuating the cycle of socioeconomic and health inequalities [14,17,18,19].

While nutrition plays a key role in preventing child stunting, other risk factors have been identified, such as IUGR and low birthweight, childhood recurrent infections, maternal short stature and underweight, household low SES and food insecurity, higher number of children aged <5 years per household, lack of access to healthcare and education, and contextual factors mostly related to unimproved safe water, sanitation, and hygiene (WASH) systems [3,8]. Therefore, stunting emerges from complex multidimensional and multilevel risk factors, which are presented by the WHO model on stunting. This model, developed by an experts’ committee, depicts how distal factors (e.g., community and societal factors) influence proximal factors (e.g., household characteristics and maternal behaviors) and how these factors impact childhood linear growth, ultimately producing stunting and its related comorbidities with short- and long-term consequences affecting individuals across their life span [3].

Nutrition is one of the key factors to achieve adequate child growth and development along with additional individual, household, and contextual factors [2]. In particular, breastfeeding has been associated with multiple maternal and child health benefits [20]. Among breastfeeding’s benefits, studies across LMICs have reported a reduction in the risk of child undernutrition, with evidence for a dose-response relationship between breastfeeding duration and reduced risk [8,21,22,23]. According to a systematic review analyzing risk factors for child stunting in 137 LMICs, when compared to other regions worldwide, the Latin American region, including Mexico, displayed a higher proportion of child stunting that was attributable to discontinued breastfeeding, which was defined as any breastfeeding <6 months among children aged ≥6 months [8]. In Mexico, breastfeeding initiation and median duration of any breastfeeding remained stable from 2006 (90.4%, 10.4 months) to 2012 (93.7%, 10.2 months); however, in 2012 Mexico registered the lowest national prevalence of exclusive breastfeeding in the past years in children aged <6 months (14.4%, 7.9 percentage points lower than in 2006), with the largest gap (18.4 percentage points lower than in 2006) observed in indigenous population in the low SES tertile living in the southern rural region [24]. Breastfeeding is particularly relevant in LMICs where contextual factors such as limited or lack of access to safe WASH systems may leave children exposed to non-innocuous complementary liquids and foods [8,20]. These exposures may increase the risk for diarrhea or other infectious diseases, which have been previously associated with increased risk for stunting [8]. This is particularly worrisome among indigenous and marginalized communities in the southern rural region of Mexico where WASH systems remain unimproved, exclusive breastfeeding is disproportionately decreasing, and the prevalence of stunting is higher when compared to the national estimates [24].

Theoretically grounded on the WHO model on stunting and from a life course perspective, it was relevant to examine the association between breastfeeding and stunting in Mexican children aged 6–35 months, while sequentially controlling for previously identified individual, household, and area risk factors. We hypothesized that children who were breastfed for ≥6 months would have lower risk for stunting when compared to those who were never breastfed. We also hypothesized that the effect of breastfeeding on stunting would vary by SES given that resources, contextual factors, and public services may vary across different SES settings [3,6]. This study contributes to advancing the knowledge base by analyzing a nationally and regionally representative sample of Mexican children in rural and urban settings. We used previously identified risk factors for stunting in other LMICs with an emphasis on breastfeeding for ≥6 months, which is considered a modifiable maternal health behavior. To our knowledge, this is the first study to use this approach within a Mexican context, contributing to the growing evidence across LMICs. Findings from this study could provide information that aids policy makers, researchers, and healthcare professionals in Mexico to develop, adapt, or modify social welfare programs, interventions, and policies that help reduce child stunting.

## Methods

The Boston College Institutional Review Board considered this protocol exempt because it is a secondary analysis of data from the 2012 Mexican National Health and Nutrition Survey (ENSANUT for its acronym in Spanish), which are de-identified and publicly available [24]. ENSANUT 2012 is a nationally representative cross-sectional survey planned and executed by the National Institute of Public Health in Mexico, which collected data on 50,528 households obtained from the Mexican Census using a probabilistic, multistage, stratified random sample [24]. This methodology allowed representativeness of rural and urban areas at national level and among 4 regions of Mexico. While child feeding data were collected from a subset of randomly selected women within these households (N = 6,254), a subsample of women had additional information collected on child birthweight and maternal characteristics, including anthropometry data, tobacco use, parity, delivery mode, and diabetes. As these factors have been associated with child stunting, we focused our analysis on this subsample. We further excluded children from analyses by using subpopulation commands if they were aged <6 months because they did not meet the exposure-of-interest criteria (i.e., any breastfeeding for ≥6 months), if they had missing or biologically implausible anthropometry data, if they were multiples or cared for by caregivers or grandmothers rather than children’s mothers, and if maternal anthropometry data were missing. We excluded multiples because they are less likely to be breastfed than singletons [25]. This criteria led to our final curated subsample, which included individual, household, and area risk factors for child stunting on 2,089 singleton children aged 6–35 months.

We assessed whether our final subsample differed from the larger sample, which included data on breastfeeding and child overweight but were missing relevant data such as child birthweight and maternal anthropometry data, as well as whether sampling weights (as provided for children in the infant feeding dataset) needed to be recalculated. In order to do so, we analyzed data using two strategies. First, we generated an indicator variable and ran a logistic regression model comparing those who would be included and excluded in our subset. Second, and following analytical recommendations from ENSANUT experts, we ran weighted and unweighted percentages and means to compare whether the whole infants sample and the subset were comparable in terms of distribution of the main independent and dependent variables. We found no significant differences, and these two strategies led us to conclude that the exclusions did not lead to significant biases in the final subset and it was unnecessary to recalculate the children’s sampling weights.

### Measures

#### Breastfeeding

During the child feeding interview, women were asked, ‘Did you ever breastfeed your child? If so, do you still breastfeed? If not, for how long did you breastfeed?’ From this information, we generated a 3-category breastfeeding duration variable: never breastfed, any breastfeeding for <6 months, and any breastfeeding for ≥6 months. Any breastfeeding was defined as receiving exclusive, predominant, or partial breastfeeding or breastmilk (i.e., child received at least some breastmilk). We also examined any breastfeeding for ≥1 and ≥3 months as well as exclusive breastfeeding (i.e., child received only breastmilk) for ≥1, ≥3, and 6 months and found no significant associations (results not shown).

#### Individual factors

Child factors include age in months as continuous, sex, delivery mode (vaginal or Cesarean-section), introduction of liquids different than breastmilk <3 days postpartum, introduction of complementary foods <6 months, and birthweight, which was categorized as normal when 2.5–4 kg, low <2.5 kg, and high >4 kg according to WHO criteria. Maternal factors were age in years as continuous, educational attainment (≤ primary, some or complete secondary, some or complete high school, some college or more), having a partner or not, parity (number of live births as continuous), any type of self-reported diabetes, current tobacco use, and employment status, which was defined as follows: full-time employment was defined as working at least 40 hours per week during the past week, and formality was defined as having a paid job with contributory social protection systems [26]. Both employment status and formality were combined into a 5-category variable (not working, part-time informal, part-time formal, full-time informal, and full-time formal). We estimated maternal body mass index (BMI) from measured weight and height by ENSANUT at the time of the interview and categorized women according to WHO criteria as underweight when <18.5 kg/m^2^, normal weight 18.5 to 24.9 kg/m^2^, overweight 25 to 29.9 kg/m^2^, and obesity ≥30.0 kg/m^2^. Additionally, we included maternal height as an independent variable because there is evidence for a strong association with stunting across LMICs but particularly in Latin American countries, such as Guatemala, Honduras, El Salvador, Ecuador, Perú, and Bolivia, in which the fraction of child stunting attributable to short maternal stature is higher when compared to other regions [8,19]. Moreover, short stature in Mexican women has been associated with lower SES, lower educational attainment, and greater marginalization when compared to taller counterparts. We further classified maternal height as short stature if it was ≤148.5 cm, which corresponds to the lowest quartile for adult women in Mexico [6].

#### Household factors

Number of children aged <5 years per household was included as a continuous variable given that scholars have identified a positive association with stunting [27]. Having a grandparent living in the same household was identified and included as a dichotomous measure in the analysis given the evidence that they may influence children’s health outcomes including weight status [28]. SES was estimated by ENSANUT through principal component analysis, using household conditions, total number of people living in the household, basic household infrastructure, and number of domestic appliances to categorize households in tertiles as low, medium, and high. Measurement of food security was estimated by ENSANUT using an adapted 15-item questionnaire of the Latin American and Caribbean Food Security Scale, which categorized households as secure and insecure mild, moderate and severe [24]. We re-categorized this into 3 groups by collapsing moderate and severe insecurity. Additionally, we included drainage type as a proxy for WASH systems given that these factors have been strongly associated with stunting in LMICs [8,29]. Drainage system included sewer, septic, and other types of systems that were mostly described as house-made structures draining into close-by land or waterbody sources.

#### Area factors

These were 4 regions in Mexico (i.e., north, central, metropolitan (Mexico City), and south) and communities’ population size (according to the survey’s design, urban was defined as community population size of ≥2,500 individuals and rural as <2,500) [24]. These two were combined into a 7-category regions variable (north-urban, north-rural, center-urban, center-rural, Mexico City-urban, south-urban, and south-rural).

#### Child stunting

Trained and standardized interviewers from ENSANUT measured length/height following age-pertinent protocols to reduce systematic errors and registered age at measurement [24]. We analyzed anthropometry from raw data provided by ENSANUT using the STATA macro from the WHO growth standards. Our outcome variable was stunting defined as length/height-for-age, for sex, under –2 SD of the WHO child growth standards median referred to as LfA-z-score, and data flagged as biologically implausible were excluded (LfA-z-score <–6 or >6) [2].

### Analytical Approach

We computed a series of analyses to examine the associations between breastfeeding duration, individual, household, and area factors with child stunting. Frequencies, weighted percentages and means, Pearson’s chi-square tests, and unadjusted logistic regression models were used to examine bivariate associations. We then assessed the association between child stunting and breastfeeding duration first in a bivariate model and then controlling for individual, household, and area factors using sequential stepwise logistic regression models (Models 1–4). In the fully-adjusted model 4, we tested an interaction to assess whether the association between stunting and breastfeeding duration differed by SES. The interaction was not significant (p ≥ 0.05) and results are not shown. We estimated the variance inflation factors for each adjusted model to test for high intercorrelations between the independent variables and found no evidence for multicollinearity problems.

While model 4 (fully adjusted) included a fixed effect by area factors allowing to compare the odds ratios for child stunting by areas-regions, it did not account for the multilevel structure in the subset. This means that we have one maternal-child dyad per household (level-1, N = 2,089) nested within areas-regions in Mexico (level-2, N = 7). Multilevel modelling would account for the fact that child-mother dyads from a given area-region share a frame of reference and that there may be differences between areas-regions. Consequently, we computed model 5 using a mixed-effects 2-level logistic regression analysis to account for area factors’ variance and tested the association between breastfeeding duration and child stunting while allowing a random intercept by area factors.

Data were analyzed using the statistical package STATA SE version 15.1 (STATA Corporation, Texas, U.S), and survey commands were used to account for children sampling weights, primary sampling units, and strata following ENSANUT analytic guidelines.

## Results

In this subsample, 94.3% of Mexican children initiated breastfeeding, 71.1% received any breastfeeding for ≥6 months, and 12.3% were stunted. These and all other descriptive data are presented in Table 1.

**Table 1:** Descriptive statistics (weighted percentages with frequencies and weighted means with standard deviations (SD)) and unadjusted odds ratios (UOR) with 95% confidence intervals (CI) of risk factors for stunting among Mexican children aged 6–35 months (N = 2,089).

	Subsample	Stunting	

	% (n)/mean (SD)	% (n)/mean (SD)	UOR (95% CI)

**Overall**		12.3 (238)	
**Individual factors**			
**Child**			
Age (months)	21.1 (8.7)	22.0 (8.0)	1.01 (0.99–1.03)
Sex			
Male	55.2 (1,106)	14.4 (137)	1
Female	44.8 (983)	9.7 (101)	0.63 (0.44–0.91)*
Birthweight			
Low	9.6 (172)	28.2 (49)	3.19 (1.79–5.69)***
Normal	84.3 (1,791)	11.0 (180)	1
High	6.1 (126)	5.1 (9)	0.44 (0.18–1.04)
Delivery mode			
Vaginal	56.5 (1,226)	13.4 (159)	1
Cesarean-section	43.5 (863)	10.9 (79)	0.79 (0.54–1.16)
Breastfeeding duration			
Never breastfed	5.7 (125)	20.2 (19)	1
Any breastfeeding <6 months	23.2 (433)	7.7 (28)	0.33 (0.13–0.81)*
Any breastfeeding ≥6 months	71.1 (1,531)	13.1 (191)	0.60 (0.29–1.23)
Liquids ≠ than breastmilk ≤3 days postpartum			
No	55.9 (1,128)	13.8 (135)	1
Yes	44.1 (961)	10.3 (103)	0.72 (0.48–1.07)
Complementary foods <6 months			
No	36.7 (837)	11.8 (90)	1
Yes	63.3 (1,252)	12.6 (148)	1.49 (1.03–2.13)*
**Maternal**			
Age (years)	27.9 (6.6)	28.7 (6.1)	1.02 (0.99–1.05)
Employment			
Not working	67.5 (1,481)	12.8 (177)	1
Part-time informal	6.4 (120)	15.1 (12)	1.21 (0.47–3.15)
Part-time formal	10.2 (195)	10.3 (21)	0.78 (0.39–1.53)
Full-time informal	9.6 (188)	9.5 (19)	0.72 (0.35–1.45)
Full-time formal	6.2 (105)	11.2 (9)	0.85 (0.33–2.19)
Education			
≤ Primary	25.3 (596)	16.8 (96)	2.17 (1.16–4.04)*
Some secondary or secondary	41.3 (904)	13.2 (102)	1.62 (0.89–2.95)
Some high school or high school	21.0 (388)	8.5 (32)	1
Some college or >	12.4 (201)	6.5 (8)	0.75 (0.24–2.33)
Partner status			
No	15.7 (312)	11.3 (33)	1
Yes	84.3 (1,777)	12.5 (205)	0.89 (0.51–1.55)
Ethnicity			
Non-indigenous	76.9 (1,539)	10.5 (136)	1
Indigenous	23.1 (550)	18.2 (102)	1.89 (1.27–2.81)**
Parity (number of live births)	2.4 (1.4)	2.7 (1.5)	1.19 (1.08–1.32)**
BMI			
Underweight	2.7 (60)	10.8 (8)	0.74 (0.26–2.13)
Normal weight	38.8 (749)	14.0 (98)	1
Overweight	34.9 (770)	11.2 (82)	0.78 (0.48–2.13)
Obesity	23.6 (510)	11.2 (50)	0.77 (0.46–1.29)
Height (cm)			
(Short stature) ≤ 148.5	19.0 (432)	27.7 (113)	3.57 (2.34–5.45)***
148.6 – 157.8	49.5 (1,047)	9.7 (95)	1
≥ 157.9	31.5 (610)	7.1 (30)	0.71 (0.39–1.27)
Self-reported diabetes (any type)			
No	96.2 (2,018)	12.0 (230)	1
Yes	3.7 (71)	20.5 (8)	1.89 (0.71–5.06)
Current tobacco use			
No	87.3 (1,907)	12.1 (216)	1
Yes	12.7 (182)	13.6 (22)	1.14 (0.59–2.22)
**Household factors**			
Number of children aged <5 years	1.4 (0.6)	1.5 (0.6)	1.43 (1.02–1.99)*
Grandparent(s) cohabiting			
No	69.2 (1,484)	13.8 (181)	1
Yes	30.8 (605)	8.8 (57)	0.60 (0.38–0.93)*
Socioeconomic status			
Low	38.5 (898)	16.9 (135)	1.83 (1.18–2.82)**
Medium	34.1 (730)	10.0 (70)	1
High	27.4 (461)	8.7 (33)	0.86 (0.45–1.64)
Food Security			
Secure	25.7 (491)	8.3 (39)	1
Mild Insecure	42.4 (940)	10.5 (97)	1.30 (0.77–2.21)
Moderate to Severe Insecure	31.9 (658)	17.9 (102)	2.42 (1.41–4.13)**
Drainage system			
Sewer	69.7 (1,235)	11.2 (119)	1
Septic	22.3 (640)	13.1 (81)	1.19 (0.80–1.79)
Other	7.9 (214)	19.4 (38)	1.90 (1.09–3.31)*
**Area factors**			
Region/Area Density			
North/Urban	16.1 (321)	8.5 (29)	1
North/Rural	3.2 (125)	6.9 (8)	0.79 (0.29–2.17)
Center/Urban	21.6 (454)	9.3 (39)	1.10 (0.57–2.09)
Center/Rural	11.0 (324)	8.7 (27)	1.02 (0.52–2.03)
Metropolitan/Urban	16.0 (88)	18.5 (15)	2.43 (1.17–5.02)*
South/Urban	18.0 (420)	9.9 (46)	1.18 (0.66–2.10)
South/Rural	14.1 (357)	21.2 (74)	2.89 (1.67–4.98)***

BMI: body mass index. * p < 0.05, ** p < 0.01, *** p < 0.001.

According to bivariate analyses, individual protective factors against child stunting were being female (unadjusted odds ratio (UOR) 0.63, 95% confidence interval (CI) 0.44–0.91) and receiving any breastfeeding for <6 months (UOR 0.33, 95% CI 0.13–0.81). In contrast, individual risk factors for child stunting were low birthweight (UOR 3.19, 95% CI 1.79–5.69), introduction of complementary foods <6 months (UOR 1.49, 95% CI 1.03–2.13), low maternal education (≤primary) (UOR 2.17, 95% CI 1.16–4.04), mothers self-identifying as indigenous (UOR 1.89, 95% CI 1.27–2.81), higher parity (UOR 1.19, 95% CI 1.08–1.32), and maternal short stature (UOR 3.57, 95% CI 2.34–5.45). Regarding household factors, grandparents cohabiting were protective against child stunting (UOR 0.60, 95% CI 0.38–0.93). In contrast, household risk factors for child stunting were higher number of children aged <5 years (UOR 1.43, 95% CI 1.02–1.99), low SES (UOR 1.83, 95% CI 1.18–2.82), moderate to severe household food insecurity (UOR 2.42, 95% CI 1.41–4.13), and having other type of drainage systems (UOR 1.90, 95% CI 1.09–3.31). Regarding area factors, when compared to living in the north-urban region, children living in the Metropolitan area (i.e., Mexico City) and in the south-rural region were at higher risk for stunting (UOR 2.43, 95% CI 1.17–5.02; 2.89, 1.67–4.98, accordingly) (Table 1).

We found evidence for consistent risk and protective factors for child stunting across models (Table 2). When compared to never breastfed and holding all other variables constant, a consistent protective factor against child stunting was any breastfeeding for <6 months, with similar direction and effect size across all models; likewise, any breastfeeding for ≥6 months had similar direction but the CI included the null value except in the fully adjusted model 4 (adjusted odds ratio (AOR) 0.45, 95% CI 0.20–0.99). Being female was an additional protective factor against child stunting identified in models 2–4. Compared to their corresponding reference groups and holding all other variables constant, consistent risk factors for stunting across all models were child’s low birthweight, maternal short stature, higher number of children aged <5 years per household, and moderate to severe household food insecurity. Additional risk factors were found in models 4 and 5. Any type of maternal self-reported diabetes was a risk factor for child stunting only in model 4 (AOR 2.50, 95% CI 1.05–5.92); and in model 5, older children (AOR 1.02, 95% CI 1.01–1.04), indigenous mothers (AOR 1.49, 95% CI 1.07–2.06), and mothers with current tobacco use (AOR 1.92, 95% CI 1.13–3.26) were at higher odds for child stunting.

**Table 2:** Models (odds ratios and 95% confidence intervals) to assess the association between breastfeeding duration and individual, household, and area factors, with stunting in Mexican children aged 6–35 months (N = 2,089).

	Model 1	Model 2	Model 3	Model 4	Model 5

**Bf duration** (Ref. NBF)					
Any breastfeeding <6 months	0.33 (0.13–0.81)*	0.36 (0.15–0.90)*	0.32 (0.13–0.81)*	0.32 (0.13–0.78)*	0.47 (0.24–0.94)*
Any breastfeeding ≥6 months	0.60 (0.29–1.23)	0.52 (0.24–1.13)	0.46 (0.20–1.06)	0.45 (0.20–0.99)*	0.68 (0.37–1.24)
**Individual factors**					
**Child**					
Age (months)		1.01 (0.99–1.03)	1.01 (0.99–1.03)	1.01 (0.99–1.03)	1.02 (1.01–1.04)*
Sex (Ref. Male) Female		0.58 (0.38–0.87)*	0.56 (0.37–0.85)**	0.56 (0.37–0.85)**	0.84 (0.63–1.13)
Birthweight (Ref. Normal & High)					
Low		3.05 (1.64–5.65)***	3.14 (1.71–5.78)***	2.91 (1.60–5.29)***	3.21 (2.15–4.79)***
Delivery mode (Ref. Vaginal)					
Cesarean-section		0.84 (0.55–1.27)	0.83 (0.54–1.28)	0.82 (0.53–1.26)	0.74 (0.53–1.01)
Liquids ≠ than breastmilk ≤3 days postpartum (Ref. No)					
Yes		0.80 (0.51–1.23)	0.81 (0.53–1.25)	0.87 (0.58–1.32)	1.00 (0.73–1.37)
Complementary foods <6 months (Ref. No)					
Yes		1.28 (0.85–1.93)	1.38 (0.91–2.10)	1.38 (0.90–2.13)	1.32 (0.97–1.80)
**Maternal**					
Age (years)		1.01 (0.97–1.05)	1.01 (0.97–1.06)	1.01 (0.97–1.06)	1.01 (0.98–1.04)
Employment (Ref. Not working)					
Part-time informal		1.60 (0.67–3.80)	1.76 (0.79–3.90)	1.77 (0.84–3.72)	1.19 (0.60–2.34)
Part-time formal		0.79 (0.39–1.58)	0.88 (0.44–1.75)	0.90 (0.45–1.83)	0.90 (0.53–1.51)
Full-time informal		1.29 (0.57–2.94)	1.62 (0.72–3.64)	1.64 (0.72–3.73)	1.43 (0.81–2.51)
Full-time formal		0.99 (0.38–2.56)	1.12 (0.44–2.87)	1.05 (0.42–2.65)	0.93 (0.44–1.98)
Education (Ref. High school)					
≤ Primary		1.80 (0.88–3.66)	1.60 (0.78–3.26)	1.62 (0.79–3.35)	1.39 (0.85–2.28)
Some secondary or secondary		1.91 (1.08–3.31)*	1.86 (1.04–3.33)*	1.92 (1.08–3.45)	1.41 (0.90–2.22)
Some college or >		1.02 (0.35–2.95)	1.02 (0.36–2.95)	1.04 (0.37–2.94)	0.61 (0.26–1.44)
Partner status (Ref. Yes) No		0.88 (0.53–1.46)	0.94 (0.57–1.54)	0.95 (0.58–1.56)	0.95 (0.60–1.52)
Ethnicity (Ref. Non-indigenous)					
Indigenous		1.30 (0.83–2.04)	1.28 (0.81–2.04)	1.26 (0.78–2.03)	1.49 (1.07–2.06)*
Parity (number of live births)		1.06 (0.90–1.26)	0.95 (0.79–1.15)	0.95 (0.79–1.15)	0.98 (0.86–1.13)
BMI (Ref. Normal weight)					
Underweight		0.69 (0.20–2.31)	0.77 (0.21–2.79)	0.76 (0.20–2.84)	0.85 (0.36–2.03)
Overweight		0.61 (0.37–0.99)*	0.64 (0.39–1.03)	0.64 (0.40–1.04)	0.68 (0.48–0.96)*
Obesity		0.62 (0.36–1.06)	0.63 (0.37–1.08)	0.65 (0.38–1.11)	0.64 (0.43–0.96)*
Height (cm) (Ref. 148.6-157.8)					
(short stature) ≤ 148.5		3.58 (2.21–5.80)***	3.59 (2.23–5.77)***	3.46 (2.14–5.61)***	3.16 (2.26–4.42)***
≥ 157.9		0.73 (0.42–1.28)	0.70 (0.40–1.22)	0.71 (0.41–1.24)	0.56 (0.36–0.87)*
Diabetes (any type) (Ref. No) Yes		2.31 (0.90–5.95)	2.48 (1.00–6.17)	2.50 (1.05–5.92)*	1.34 (0.61–2.98)
Current tobacco use (Ref. No) Yes		1.97 (0.96–4.04)	2.00 (0.97–4.13)	1.88 (0.94–3.77)	1.92 (1.13–3.26)*
**Household factors**					
Number of children aged <5 years			1.60 (1.09–2.35)*	1.59 (1.09–2.30)*	1.47 (1.11–1.97)**
Grandparent(s) cohabiting (Ref.No)					
Yes			0.65 (0.42–1.01)	0.66 (0.43–1.03)	0.83 (0.57–1.21)
Socioeconomic status (Ref. Medium)					
Low			1.08 (0.64–1.79)	1.02 (0.61–1.70)	1.05 (0.73–1.50)
High			1.05 (0.54–2.04)	0.99 (0.52–1.89)	1.03 (0.63–1.67)
Food Security (Ref. Secure)					
Mild Insecure			1.39 (0.79–2.45)	1.36 (0.77–2.38)	1.16 (0.76–1.76)
Moderate to Severe Insecure			2.24 (1.23–4.07)**	2.16 (1.20–3.90)*	1.58 (1.02–2.46)*
Drainage system (Ref. Sewer)					
Septic			0.94 (0.59–1.52)	0.93 (0.54–1.59)	0.93 (0.65–1.32)
Other			1.14 (0.56–2.34)	1.15 (0.51–2.59)	1.06 (0.66–1.71)
**Area factors**					
Region/Area Density (Ref. N/U)					
North/Rural				0.66 (0.22–2.01)	
Center/Urban				0.92 (0.46–1.84)	Variance
Center/Rural				0.85 (0.38–1.90)	5.82^–33^ (SE 1.34^–17^)
Metropolitan/Urban				1.55 (0.75–3.24)	ICC 1.77^–33^
South/Urban				0.88 (0.43–1.82)	(SE 4.06^–18^)
South/Rural				1.44 (0.70–2.94)	

Bf: breastfeeding, Ref: reference group, NBF: never breastfed, BMI: body mass index, N/U: north urban area, SE: standard error, ICC: intraclass correlation coefficient. Model 1 examined the association between breastfeeding and child stunting without adjusting for any of the covariates; models 2 through 4, sequentially, adjusted for individual, household, and area factors. Model 5 examined the association between breastfeeding and child stunting using a 2-level logistic approach. * p < 0.05, ** p < 0.01, *** p < 0.001.

In the model 5 (multilevel mixed-effects model), level-2 variance was 5.82^–33^ (standard error (SE) 1.34^–17^) with an intraclass correlation coefficient (ICC) of 1.77^–33^ (SE 4.06^–18^).

## Discussion

Among a nationally representative subsample of children in Mexico, we found that in 2012, 12.3% of children aged 6–35 months were stunted. We found evidence for a protective effect of breastfeeding on stunting when compared to those who were never breastfed. There was no differential effect of breastfeeding on stunting by household SES as we had hypothesized. We were able to confirm previously identified risk factors, which have been described in the literature, such as child low birthweight, mother self-identifying as indigenous, maternal short stature, families with higher number of children aged <5 years, and moderate to severe household food insecurity.

Coinciding with previous studies in LMICs, including countries in Latin America, Asia, and Sub-Saharan Africa, our results have shown that children who initiate breastfeeding (any breastfeeding for < or ≥6 months) were at lower risk for stunting [8,21,30]. This association has been mainly explained at the individual level by the breastmilk’s immune-protective factors, which help strengthen the child immature immune system, reducing diarrheal episodes and other infectious diseases, which have been identified as leading risk factors for stunting, as well as reduced exposure to non-innocuous complementary liquids or foods, such as unsafe drinking water [8,20,31]. Similarly, in agreement with several scholars, we identified a lower risk for females to be stunted [21,27,32]. There is no consensus or clear mechanism for this association; however, it may be partially explained through maternal fetal environment and differential growth trajectories by gender. In the uterus, male fetuses invest greater resources in growth being at a higher risk of becoming undernourished and eventually being born with low birthweight and plausible linear growth failure [33].

We identified low birthweight and maternal short stature to be consistent individual-level risk factors for stunting. Low birthweight typically resulting from IUGR has been previously identified as one of the leading risk factors with the highest attributable burden of stunting across LMICs [8,21,34]. However, it is worth emphasizing that in our subsample we could not identify whether low birthweight was due to IUGR. Maternal short stature was associated with higher odds for stunted offspring, regardless of SES. This supports the intergenerational effect of early undernutrition, which is intertwined with lower living conditions and the widening of health, social, and economic disparities. Using nationally-representative data, scholars reported that women with short stature in Mexico (i.e., height ≤ 148.5 cm) were identified in the most vulnerable groups: low SES and education and greater marginalization—shared conditions with the indigenous population [6,35]. We found that mothers self-identifying as indigenous were at higher risk for stunted offspring in the bivariate and multilevel analyses, and this has been previously reported in Mexico [10]. The social, health, and economic gaps that indigenous communities in Mexico face have been consistently reported, and such gaps widen even more for indigenous women [35]. Additionally, in our subsample, the highest proportion of short stature women was found among indigenous women, which may further aggravate child stunting outcomes. Recent genetic analyses have identified idiopathic short stature among the Mexican indigenous population across generations [36]. While this trait may partially explain offspring’s short stature in this population, it does not fully account for the persistence of the intergenerational effect of undernutrition and its negative cognitive and developmental outcomes, which continue to be pervasive in this group in Mexico.

At the household level, consistent risk factors for child stunting were higher number of children aged <5 years and moderate to severe food insecurity. Scholars have previously described that a higher number of children aged <5 years per household may be associated with sub-optimal breastfeeding practices among younger siblings, as well as competition for food and other resources, which may ultimately lead to child undernutrition [21,27]. Food insecurity has been a strong predictor for child stunting in Mexico and other LMICs where children’s diets face qualitative and quantitative deficiencies resulting in child undernutrition over time [37,38].

Several efforts have been implemented on a global scale to prevent and end early undernutrition, including child stunting. According to a systematic review, conditional cash transfers (CCT) in Latin America have shown to be effective against child stunting by addressing access to healthcare, maternal and child nutrition, and immunization coverage [39]. Other strategies targeting individual-level factors, such as nutritional interventions, have also been shown to be protective [40]. However, CCT programs or nutritional interventions may not mitigate child stunting if they are isolated from other systems, such as food or WASH systems. In Mexico, for the past decades, the main anti-poverty strategy of the federal government was a CCT program currently referred to as Prospera, which offered cash transfers to the poorest families conditional on regular school attendance and family healthcare visits as well as provision of nutritional supplements for pregnant and lactating women and their children. Overall, this program had helped improve child health and developmental outcomes [41]; however, in 2019, Mexico’s elected federal government, installed in December 2018, cancelled the program and prioritized 30 other social welfare programs and projects [42]. According to the available information, none of these prioritized programs directly support the nutritional status of women with children aged <2 years. However, some of these programs support overall well-being using diverse strategies, and it will be fundamental to monitor and evaluate the impact of these social policies on child undernutrition, including stunting.

From an analytical perspective, accounting for the 2-level structure in our subsample (Model 5 in Table 2), we were able to confirm associations with similar direction and effect size than those in previous models and to identify additional associations that have been previously described by other scholars. However, according to conservative rules, in order to have reliable estimates in 2-level models, the 30–30 or 50–20 criteria should be applied (i.e., ≥30 level-2 groups and ≥20 level-1 observations per group). Other less conservative scholars argue that even when these rules are not met, ignoring the multilevel structure and assuming that the group variance is zero would not be advisable [43]. While our dataset did not support the conservative criteria (i.e., we had 7 level-2 groups and 100 minimum level-1 observations per group, 7–100), we decided to compare a fixed-effects fully-adjusted model (Model 4) with a mixed-effects 2-level model with random intercept at area factors (Model 5). For comparison purposes, we computed some fit statistic tests. The likelihood ratio test versus logistic model yielded a p > 0.05 and the ICC estimate was 5.82^–33^. The first value indicates that there were no statistical differences in the estimates between models 4 and 5. The ICC value provides the variance in the model, which is explained by differences between areas-regions. While there seems to be no objective cut-off values for ICC, which ranges from 0–1, some scholars have recommended that a value ≤0.10 may indicate that a multilevel model would not be adequate, which is our case; nonetheless, most scholars have argued that this should not justify disregarding multilevel models, particularly when nesting is straightforward such as with our subsample [43].

By using ENSANUT 2012 we were able to examine diverse individual, household, and area risk factors that have been described in the WHO conceptual model for stunting. One of the strengths of this dataset is that anthropometric data for children and their mothers were not self-reported but measured by trained personnel using age-pertinent protocols. This allowed us to estimate children’s z-scores according to the WHO’s multicenter study, which included growth data from breastfed children in HICs and LMICs, as well as estimate reliable measures of maternal short stature and BMI.

The primary limitation of cross-sectional analyses is that we could not rule out reverse causation or assess the temporality of some risk or protective factors preceding child stunting. Another limitation was that breastfeeding data were collected at the time of the interview with children’s age ranging from 0–35 months. We could not use breastfeeding as a continuous variable, and we had to exclude children aged <6 months from analysis because they did not meet the exposure criteria of any breastfeeding for ≥6 months. We relied on maternal recall of child feeding practices with some cases still breastfeeding and others reporting retrospective data from weeks up to 2.5 years. Scholars who have studied respondent’s recall bias on retrospectively collected breastfeeding data suggest that studies exploring breastfeeding practices be conducted either prospectively or within <1 month following weaning [44]. We acknowledge that respondent recall bias on breastfeeding practices is likely present. We could not discern between children who were fed at the breast or those receiving breastmilk in bottles. In the latter case, improved WASH systems would play an important role in order to prevent plausible breastmilk cross-contamination. In our subset, we could not measure access to improved WASH systems, and while we used a proxy (type of drainage), it did not provide finely detailed information to be able to accurately assess WASH factors. Additionally, there were no available data on other relevant variables that have been identified as key risk factors for stunting. These include prenatal tobacco exposure (we used smoking at the time of the survey as a proxy for prenatal or pregnancy exposure), maternal nutritional status preconception and during pregnancy and lactation, IUGR (we used birthweight as a proxy), short birth spacing, prenatal and pregnancy healthcare quality, macro and micronutrient child deficiencies, among other higher-level factors that support healthy lifestyles [8]. Excluding the aforementioned risk factors may have led to underspecified models.

In conclusion, our results suggest that efforts to prevent and reduce child stunting should include pre- and post-natal components. We recommend that prenatal strategies focus on access to qualified and continued healthcare in order to prevent low birthweight, with an emphasis on communities where maternal short stature is the norm and among indigenous communities. Efforts should also focus postnatally by supporting positive maternal health behaviors, including breastfeeding initiation and continuation and innocuous complementary feeding. According to our literature review and pertinent to Mexico, in order to support these behaviors, at community and higher levels, policies and interventions should aim to enforce the International WHO Code of Marketing of Breastmilk Substitutes and local legislation to restrict hospital use of infant formula; extend paid maternity leave up to 6 months with adequate support systems that facilitate breastfeeding continuation to support women employed in the formal sector; implement a maternity cash transfer to support women employed in the informal sector; improve the training of healthcare providers to increase the quality of services provided for mothers and their children; enable food systems to provide healthy and innocuous foods; and improve or provide safe WASH systems where not yet available [10,12,20,29,31,40,45,46]. Pre- and post-natal efforts should also focus on households with moderate to severe food insecurity and in families with a higher number of children aged <5 years. While these interventions would benefit all families, efforts to end stunting should target environments with evidence of intergenerational effects of undernutrition (i.e., maternal short stature with offspring low birthweight). The multilevel risk factors identified in this paper describe the context from which child stunting emerges in Mexico, which contributes to the growing evidence across LMICs. By focusing on evidence-based data and developing pertinent interventions and policies, the maternal-child dyad may be able to thrive against stunting.

## Additional File

The additional file for this article can be found as follows:

10.5334/aogh.2836.s1 Analytic Subsample. Curated subsample from the ENSANUT 2012.
